# Safety evaluation of medroxyprogesterone acetate: a pharmacovigilance analysis using FDA adverse event reporting system data

**DOI:** 10.3389/fphar.2024.1491032

**Published:** 2024-12-11

**Authors:** Luyang Su, Ren Xu, Yanan Ren, Shixia Zhao, Weilan Liu, Zeqing Du

**Affiliations:** ^1^ Physical Examination Center, Hebei General Hospital, Shijiazhuang, China; ^2^ Department of Obstetrics and Gynecology, Hebei General Hospital, Shijiazhuang, China; ^3^ Department of Obstetrics and Gynecology, Second Hospital of Hebei Medical University, Shijiazhuang, China

**Keywords:** medroxyprogesterone acetate, real-world data analysis, adverse drug events, FAERS, unintended pregnancy

## Abstract

**Background:**

Medroxyprogesterone acetate (MPA), a synthetic progestogen, is extensively used for the treatment of various conditions, including contraception, irregular menstruation, functional uterine bleeding, and endometriosis. However, like all pharmaceutical agents, MPA is associated with adverse drug reactions. This study aimed to evaluate the adverse events (AEs) associated with MPA in by analyzing real-world data from the U.S. Food and Drug Administration’s Adverse Event Reporting System (FAERS). By providing a comprehensive assessment of the safety profile of MPA, this study seeks to support informed clinical decision-making.

**Methods:**

Data covering the period from the first quarter of 2004 to the first quarter of 2024 were collected from the FAERS database. Disproportionality analyses were conducted using several statistical methods, including reporting odds ratio (ROR), proportional reporting ratio (PRR), empirical Bayesian geometric mean (EBGM). Additionally, time-to-onset (TTO) analysis was employed to quantify the signals of the MPA-associated AEs.

**Results:**

A comprehensive dataset comprising 21,035,995 AE reports was compiled. Among these, 3,939 women reported using MPA as a contraceptive method. The reports covered 27 system organ classes (SOCs) and 25 high-frequency AE signals. Notably, significant AEs were identified, some of which were not previously detailed in the medication’s prescribing information. Unforeseen significant AEs such as unintended pregnancy (n = 623; ROR, 6.65; ROR025, 6.1; χ^2^, 2,482.38; PRR, 6.41; EBGM, 5.69; EBGM05, 5.29), bone pain (n = 35; ROR, 13.78; ROR025, 9.4; χ^2^, 311.2; PRR, 13.75; EBGM, 10.59; EBGM05, 7.69), gait disturbance (n = 34; ROR, 2.82; ROR025, 1.99; χ^2^, 37.31; PRR, 2.88; EBGM, 2.7; EBGM05, 2.02), dental caries (n = 15; ROR, 23.16; ROR025, 12.32; χ^2^, 204.26; PRR, 23.14; EBGM, 15.23; EBGM05, 8.98), decrease in blood pressure (n = 15; ROR, 3.88; ROR025, 2.29; χ^2^, 29.35; PRR, 3.88; EBGM, 3.63; EBGM05, 2.33), and osteonecrosis (n = 9; ROR, 23.44; ROR025, 10.36; χ^2^, 123.67; PRR, 23.43; EBGM, 15.35; EBGM05, 7.75) were identified as AEs that were not previously outlined in the prescribing information of the medication.

**Conclusion:**

Our findings align with clinical observations, highlighting the emergence of previously unreported AE signals associated with MPA and their demographic and TTO characteristics. Further pharmaco-epidemiological studies are required to substantiate these observations.

## 1 Introduction

Over the years, the proportion of women of reproductive age globally using modern contraceptive methods has increased to 48% (Teal and Edelman, 2021). Injectable contraception is a popular contraceptive method, especially in low-incomes countries. In the United States, nearly 3% of women of reproductive age use medroxyprogesterone acetate (MPA), and up to 26% of sexually active women have used it at some point (Upadhyay et al., 2016). Medroxyprogesterone acetate, a steroidal progestin, is the sole contraceptive injection approved by the U.S. Food and Drug Administration in October 1992. Its clinical efficacy is widely recognized, with proven applications in contraception, management of menstrual disorders, anovulatory functional uterine bleeding (Bender, 2022), and mild to moderate endometriosis. Additionally, it is frequently used as a palliative or adjuvant therapy for inoperable, recurrent, or metastatic hormone-dependent tumors, including advanced breast, endometrial (van Weelden et al., 2022), and renal cancers. In certain cases, MPA is also used to address anorexia and body weight concerns in patients with advanced tumors. Despite its therapeutic benefits, MPA use carries associated risks.

Numerous patients experience a variety of side effects after MPA use, including amenorrhea (Sims et al., 2020), weight increase (Sims et al., 2020), osteoporosis (Aderoba et al., 2023; Wang et al., 2023), hot flush (Aderoba et al., 2023), elevated blood pressure (Jiang et al., 2023), endometrial disorders (Rosenthal and McQuillan, 2021), cardiac disorders, and vaginal mucosal related infections (Dabee et al., 2023). These adverse reactions, especially those related to the cardiovascular system, are likely to increase their health risks, cause serious consequences, and reduce the quality of life and outcomes of patients (Shiferaw et al., 2021; Lopez et al., 2019; Chauvet-Gelinier and Bonin, 2017; Irigoyen et al., 2016; Kanis et al., 2018; Santoro et al., 2021). Therefore, healthcare professionals must closely monitor patient responses when using MPA to ensure the safety and efficacy of treatment.

The U.S. Food and Drug Administration’s Adverse Event Reporting System (FAERS) database detects possible associations between medications and adverse events (AEs) as part of a broader post-marketing drug safety monitoring effort (Yu et al., 2021).

Despite being a voluntary reporting system, the FAERS database still significantly reflects real-world drug events (Xuan et al., 2023). Utilizing pertinent data from the FAERS, the present study used the reporting odds ratio (ROR) and proportional reporting ratio (PRR) to quantify AE signals associated with MPA. Additional assessments and analyses were performed to identify previously undetected AEs, offering empirical data for clinical utilization and subsequent studies on MPA.

## 2 Materials and methods

### 2.1 Data source and collection

The FAERS serves as a publicly accessible repository for post-marketing safety surveillance, gathering reports of AEs from a diverse array of sources, including healthcare providers, pharmaceutical firms, patients, and other interested parties. Based on the FAERS database, we meticulously curated and standardized MPA data, ensuring the exclusion of duplicate reports and data that were irrelevant to the female population. Focusing on the decade-long period from the first quarter (Q1) of 2004 to Q1 of 2024, we amassed a comprehensive collection of adverse drug reactions (ADRs) associated with MPA. All preferred terms (PTs) across the system organ classes (SOCs) were meticulously extracted. Within the scope of this study, MPA in the FAERS database was designated as the primary suspect drug entity, facilitating an in-depth exploration of its correlation with AEs.

**FIGURE 1:**
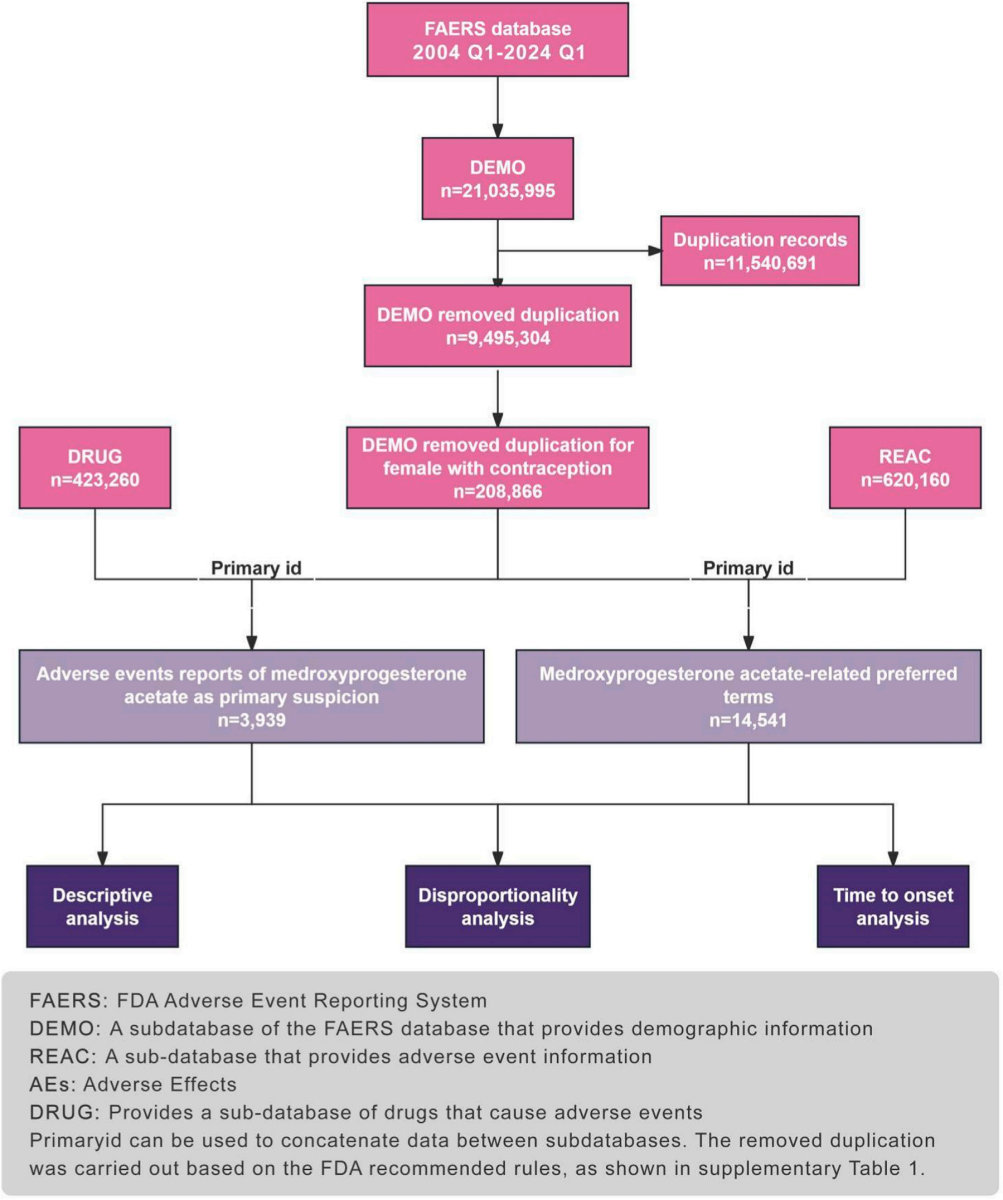
The flow diagram of selecting MPA-related AEs from the FAERS database.

This approach allows for the targeted analysis of MPA-related ADRs, providing a robust foundation for pharmacovigilance and contributing valuable insights into the safety profile of this medication. Figure 1 illustrates the study.

### 2.2 Data processing

The adverse drug events (ADEs) data collected in this study were sourced from the FAERS, established in 2004. FAERS serves as a repository for adverse event reports submitted by medical professionals, pharmaceutical companies, consumers, and others. Updated quarterly, this globally accessible and widely recognized database boasts a substantial volume of standardized data. To align with the drug market entry timeline, we retrieved reports from FAERS spanning from the first quarter of 2004 to the first quarter of 2024 and conducted data processing using R software. Within the DEMO table. clinical characteristics of patients experiencing adverse events related to MAP were compiled, including age, gender, reporting region, reporter identity, and patient outcomes. Severe adverse patient outcomes were defined as hospitalization, disability, life-threatening events, or death. We conducted preliminary data preprocessing to ensure the credibility of our findings. After eliminating duplicates and defining the population, we identified a total of 3,939 adverse event reports implicating medroxyprogesterone acetate as the primary suspect Figure 1.

### 2.3 Statistical analysis

Descriptive statistics were used to illustrate the characteristics of the AEs associated with MPA. In our study, disproportionality analysis, a common method used in pharmacovigilance, was used to identify potential associations between drugs and ADRs. Algorithms including the Reporting Odds Ratio (ROR) (Chen et al., 2024), Proportional Reporting Ratio (PRR) (Zhang et al., 2024) and empirical Bayesian geometric mean (EBGM) (Shu et al., 2021) were used to determine the statistical association between MPA and FAERS events (Supplementary Table S1). The principle of ROR and PRR is to use the classical four grid table method to compare the degree of imbalance in the proportion of AE between the target drug and other drugs. Usually, when the frequency is ≥3 and the lower limit of the 95% CI for ROR is ≥1, it indicates the detection of an ADR risk signal. If the PRR value is ≥2 and the chi square value (χ^2^) is ≥4, it indicates the detection of an ADR risk signal. EBGM method applies Bayesian discriminant theorem, which has the characteristics of high specificity, stable signal. Usually, if the 95% CI lower limit of EBGM is ≥2, it indicates the detection of ADR risk signals. This article combines three methods to screen and analyze ADR signals of MPA.

The software R (version 4.3.2) was used for all data handling and statistical analyses. Descriptive analysis was used to summarize MPA-related clinical characteristics collected from the FAERS database.

## 3 Results

### 3.1 Basic information on ADRs of MPA

Between the first quarter of 2004 and first quarter of 2024, our dataset contained 21,035,995 AE reports. Among these, 3,939 women used MPA for contraception. Details are presented in Table 1.

**TABLE 1:** Clinical Characteristics of Reports with Medroxyprogesterone acetate from the FAERS Database.

Characteristics	Number of Cases (%)
Total cases	3,939
Death cases	25 (0.6%)
Gender	
Female	3,939 (100%)
Age group (years)	
0–20	602 (15.3%)
21–40	1,924 (48.8%)
41–60	266 (6.8%)
>60	0
Unknown	1,147 (29.1%)
Reported Person	
Consumer	1,479 (37.5%)
Lawyer	96 (2.4%)
Health Professional	157 (4.0%)
Physician	474 (12.0%)
Other health-professional	916 (23.3%)
Pharmacist	380 (9.6%)
Registered nurse	6 (0.2%)
Unknow	431 (11.0%)
Reported Countries	
United States	2,490 (63.2%)
Other Countries	990 (25.1%)
Unknown	459 (11.7%)

In our cohort, individuals aged 21–40 years were disproportionately affected by AEs, representing 48.8% of all cases (n = 1924). The most serious outcome among AEs was death (n = 25; 0.6%). A substantial number of reports were sourced from the United States (n = 2,490; 63.2%). In addition, in the context of contraceptive use, the adverse reactions reported by women using MPA showed significant differences across occupational groups. The proportion of consumers was significantly high (n = 1,479; 37.5%), followed by other health professionals (n = 916; 23.3%), physicians (n = 474; 12.0%), unknown (n = 431; 11.0%), pharmacists (n = 380; 9.6%), health professionals (n = 157; 4.0%), lawyers (n = 96; 2.4%), and registered nurses (n = 6; 0.2%). However, the occupational statuses of some individuals remained unclear.

### 3.2 High frequency ADR signals of MPA

According to the judgment rules of risk signals, we have selected 25 ADR signals of MPA with a frequency of occurrence greater than 10 and sorted them, as shown in Table 2. Among them, unintended pregnancy (n = 623; ROR, 6.65; PRR, 6.41; EBGM, 5.69; EBGM05, 5.29), amenorrhea (n = 359; ROR, 2.93; PRR, 2.88; EBGM, 2.76; EBGM05, 2.52), and weight increase (n = 315; ROR, 2.55; PRR, 2.52; EBGM, 2.43; EBGM05, 2.21) were the most common adverse reactions. In addition, PTs reported with a frequency exceeding 100 occurrences included subsequent osteoporosis (n = 223), osteopenia (n = 127).

**TABLE 2:** Signal Strength of Reports of Medroxyprogesterone acetate at the Preferred Terms Level in FAERS Database.

Preferred terms	SOC	N	ROR	ROR_025_	PRR	χ^2^	EBGM	EBGM05
*Unintended pregnancy	Pregnancy, puerperium and perinatal conditions	623	6.65	6.10	6.41	2,482.38	5.69	5.29
Amenorrhoea	Reproductive system and breast disorders	359	2.93	2.63	2.88	416.23	2.76	2.52
Weight increased	Investigations	315	2.55	2.27	2.52	274.07	2.43	2.21
Osteoporosis	Musculoskeletal and connective tissue disorders	223	377.28	249.45	371.51	8,309.9	38.35	27.13
Osteopenia	Musculoskeletal and connective tissue disorders	127	190.56	126.53	188.91	4,289.23	34.94	24.81
Arthralgia	Musculoskeletal and connective tissue disorders	99	2.62	2.13	2.60	92.36	2.51	2.12
Pruritus	Skin and subcutaneous tissue disorders	88	2.64	2.12	2.63	83.51	2.53	2.11
Infertility female	Reproductive system and breast disorders	54	5.57	4.19	5.55	178.01	178.01	3.95
Hot flush	Vascular disorders	50	3.28	2.46	3.27	73.31	3.11	2.44
Nervousness	Psychiatric disorders	47	3.42	2.54	3.41	74.08	3.23	2.52
Bone density decreased	Investigations	45	156.67	82.86	156.18	1,460.89	33.67	19.76
*Bone pain	Musculoskeletal and connective tissue disorders	35	13.78	9.40	13.75	311.20	10.59	7.69
*Gait disturbance	General disorders and administration site conditions	34	2.82	1.99	2.82	37.31	2.70	2.02
Blood pressure increased	Investigations	28	3.01	2.05	3.01	34.97	2.87	2.08
Night sweats	Skin and subcutaneous tissue disorders	26	3.26	2.18	3.25	37.63	3.09	2.21
Osteoporotic fracture	Musculoskeletal and connective tissue disorders	21	875.89	117.81	874.63	833.00	40.71	7.60
Foot fracture	Musculoskeletal and connective tissue disorders	16	12.59	7.19	12.57	130.94	9.89	6.19
*Dental caries	Gastrointestinal disorders	15	23.16	12.32	23.14	204.26	15.23	8.98
*Blood pressure decreased	Investigations	15	3.88	2.29	3.88	29.35	3.63	2.33
Ankle fracture	Injury, poisoning and procedural complications	13	13.89	7.42	13.88	116.57	10.66	6.30
Osteoarthritis	Musculoskeletal and connective tissue disorders	13	8.47	4.66	8.46	71.09	7.20	4.37
Thyroid disorder	Endocrine disorders	13	4.79	2.70	4.79	34.99	4.40	2.72
Weight fluctuation	Metabolism and nutrition disorders	12	6.58	3.58	6.58	49.01	5.82	3.49
Endometrial disorder	Reproductive system and breast disorders	10	11.58	5.74	11.57	75.57	9.27	5.16
Cardiac disorder	Cardiac disorders	10	3.69	1.93	3.69	17.98	3.47	2.02

N, number of reported frequency; ROR, reporting odds ratio; ROR_025_^,^ the lower limit of 95% CI, of ROR; PRR, proportional reporting ratio; χ^2^, chi-squared; EBGM, empirical Bayesian geometric mean; EBGM, 05, the lower limit of 95% CI, of EBGM. *: New ADR, signals not mentioned in the label.

The SOC corresponding to high-frequency ADR signals is also listed in Table 2. And the SOC level corresponding to all ADR signals of MPA is shown in Table 3. The AEs associated with MPA affected 27 SOCs, suggesting that such AEs are widespread. Combining the two tables, the common SOCs of AEs in the statistical data are reproductive system and breast disorders (n = 1816), musculoskeletal and connective tissue diseases (n = 1,113) and investigations (n = 812), etc.

**TABLE 3:** System Organ Class (SOC) of adverse events of MPA in FAERS database.

SOC	N	SOC	N
General disorders and administration site conditions	2007	Metabolism and nutrition disorders	170
Reproductive system and breast disorders	1816	Immune system disorders	158
Injury, poisoning and procedural complications	1,355	Eye disorders	158
Psychiatric disorders	1,153	Cardiac disorders	126
Musculoskeletal and connective tissue disorders	1,113	Neoplasms benign, malignant and unspecified (incl cysts and polyps)	112
Nervous system disorders	1,086	Social circumstances	87
Pregnancy, puerperium and perinatal conditions	1,044	Endocrine disorders	71
Investigations	812	Blood and lymphatic system disorders	71
Gastrointestinal disorders	767	Renal and urinary disorders	67
Skin and subcutaneous tissue disorders	766	Surgical and medical procedures	59
Product issues	500	Hepatobiliary disorders	42
Infections and infestations	335	Ear and labyrinth disorders	40
Respiratory, thoracic and mediastinal disorders	303	Congenital, familial and genetic disorders	22
Vascular disorders	301		

N, number of reported frequency.

### 3.3 New ADR signals of MPA

Significant AEs at the PT level, which were not anticipated in the product labeling, were identified and included unintended pregnancy (n = 623; ROR, 6.65; PRR, 6.41; EBGM, 5.69; EBGM05, 5.29), bone pain (n = 35; ROR, 13.78; PRR, 13.75; EBGM, 10.59; EBGM05, 7.69), gait disturbance (n = 34; ROR, 2.82; PRR, 2.82; EBGM, 2.7; EBGM05, 2.02), dental caries (n = 15; ROR, 23.16; PRR, 23.14; EBGM, 15.23; EBGM05, 8.98), decrease in blood pressure (n = 15; ROR, 3.88; PRR, 3.88; EBGM, 3.63; EBGM05, 2.33), and osteonecrosis (n = 9; ROR, 23.44; PRR, 23.43; EBGM, 15.35; EBGM05, 7.75).

### 3.4 Time-to-onset analysis

**FIGURE 2:**
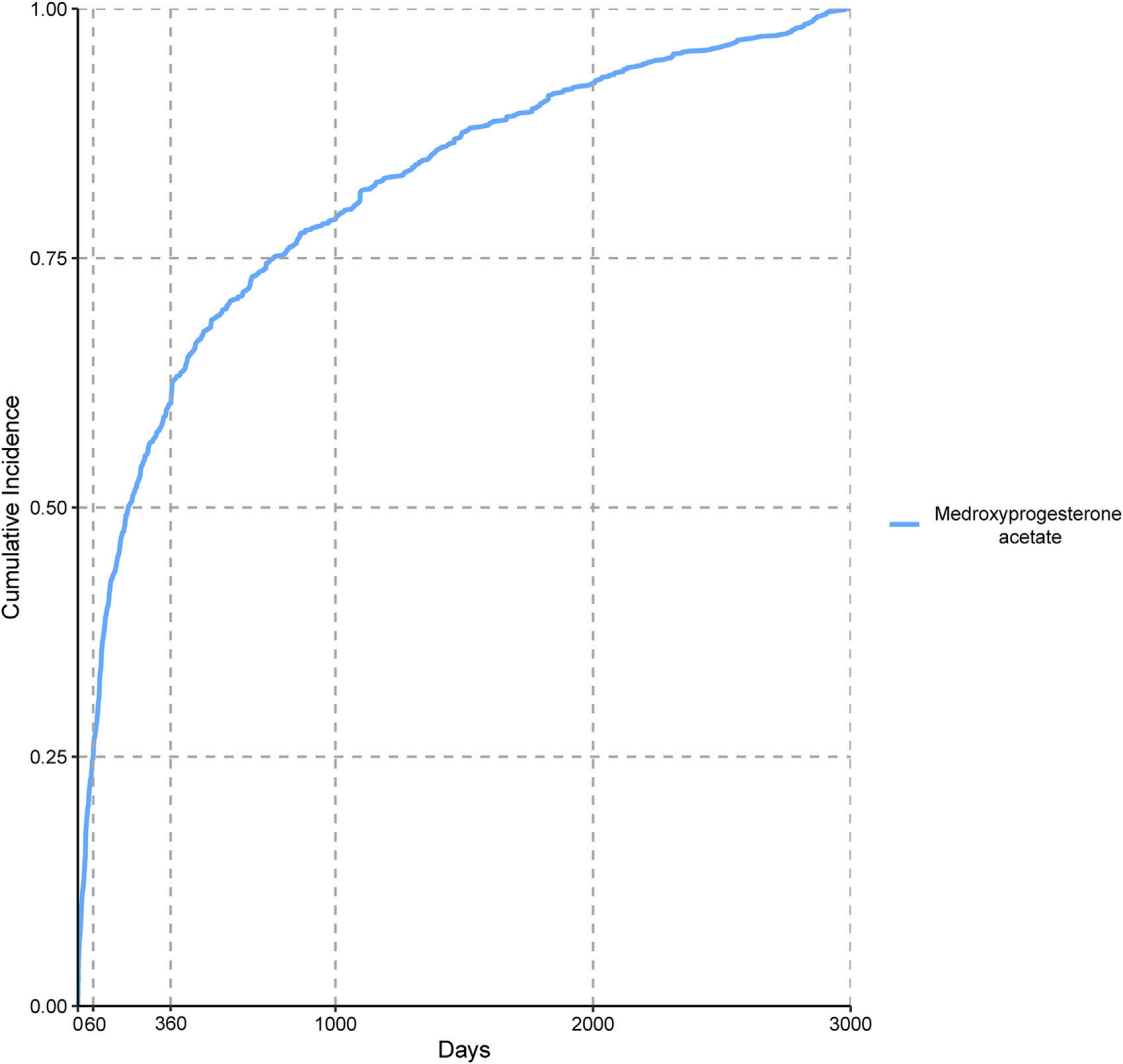
Comparison between Medroxyprogesterone acetate of AEs between patients.

We incorporated 3,939 valid cases associated with MPA in our time-to-onset analysis and the findings are presented in Table 4. The median time-to-onset of AEs associated with MPA was 195.50 d, ranging from 60 to 759.5 d, suggesting a pattern indicative of early onset. Figure 2 shows the cumulative incidence of these AEs, revealing that 25.09% occurred within the first 60 d of MPA therapy and 60.37% were reported within the first 360 d.

**TABLE 4:** Time to onset profile of adverse events in contraceptive women using Medroxyprogesterone acetate in the FAERS database.

Drug	Cases	Median(d) (25%–75%)	Shape parameter:β(95%CI)	Type
Medroxyprogesterone acetate	1,080	195.50 (60.00–759.50)	0.66 (0.63–0.69)	Early failure

CI, confidence interval.

## 4 Discussion

This study analyzed reports from the FAERS database to provide novel insights into adverse events linked to MPA. The objective of this study was to gain new insights into real-world clinical applications of MPA. Medroxyprogesterone acetate is an injectable contraceptive that inhibits ovulation and relies solely on progestins. It is highly effective and one of the most common forms of reversible contraception in the United States and worldwide (Sims et al., 2020). However, with the increased use of MPA, various uncommon AEs have continued to emerge. This study leveraged the FAERS database to mine AE reports associated with MPA by performing drug safety signal analysis at both the SOC and PT levels. Our findings revealed several noteworthy signals that were previously unacknowledged in drug labeling. Based on our current understanding, this study presents an initial real-world pharmacovigilance assessment of multiple MPA formulations. Previous results indicate that after using the drug, numerous patients experience a variety of side effects, which may include amenorrhea (Sims et al., 2020; Yu et al., 2024), osteoporosis (Cundy et al., 1991), elevated blood pressure (Aderoba et al., 2023), hot flush (Matovu et al., 2020), endometrial disease (Tamauchi et al., 2024), and heart disease (Jiang et al., 2023). Furthermore, weight change (Sims et al., 2020) is a likely reaction to the drug. In a study by Sims et al. (2020), the prevalence of obesity (defined as a BMI ≥30 kg/m^2^) among women aged 20–39 years was found to be 36.5%, based on data from the 2015–2016 National Health and Nutrition Examination Survey. These AEs correlated with our analysis of data from the FAERS database.

In the present study, the most frequent occurrence of AEs during the period Q1 of 2004–Q1 of 2024 were usually concentrated immune system, endocrine, and musculoskeletal and connective tissue disorders. Our results also showed novel and significant PTs, including unintended pregnancy, bone pain, gait disturbance, dental caries, and decreased blood pressure; however, previous clinical studies and drug documentation did not mention these adverse effects.

One adverse outcome associated with MPA use is unintended pregnancy (Watts et al., 2021), which has the largest number of cases. This phenomenon warrants further attention and may reflect several underlying issues including the following: 1. Compliance: The efficacy of contraceptive medications such as MPA is highly dependent on patient compliance. If patients fail to use MPA correctly as prescribed, either by missing injections or receiving insufficient doses, the success rate of contraception decreases, consequently increasing the risk of unintended pregnancy. 2. Method failure rates: All contraceptive methods have inherent failure rates. Even the most effective methods can fail because of various factors including drug interactions and individual variability, which can lead to contraceptive failure. 3. Lack of communication and education: Patients may be insufficiently educated about the proper use of MPA and the effectiveness of contraception, leading to misconceptions about the expected outcomes of the drug and a lack of understanding of the potential risks. To address this phenomenon, further research is necessary to identify the specific causes of the increased risk of unintended pregnancy. Subsequently, targeted measures could be implemented to enhance the success rate of contraception. These measures include improving patient education, refining medication usage guidelines, and intensifying drug interaction monitoring. In addition, it is crucial to increase awareness among healthcare professionals and patients regarding the selection and use of contraceptive methods to mitigate the risk of unintended pregnancy.

Previous studies have consistently demonstrated that MPA, a commonly used contraceptive, can reduce bone mineral density (Rosenthal and McQuillan, 2021) and increase the risk of osteoporosis. As bone density decreases, the skeletal system becomes more susceptible to fractures because of compromised bone strength. Moreover, a loss of bone mass exceeding 12% can manifest as bone pain that intensifies with rapid or severe bone loss. Osteoporosis is widely acknowledged to be intricately linked to bone loss, with the latter being a pivotal factor in the progression of the former. Bone loss, characterized by a decrease in bone mineral density, can ultimately culminate in osteoporosis (Armas and Recker, 2012). Consequently, in clinical practice, when diagnosing osteoporosis in women using MPA as a contraceptive, it is imperative to implement appropriate therapeutic interventions. These include lifestyle modifications, pharmacological treatments, and rehabilitation exercises, to alleviate pain and prevent fractures. Moreover, the role of MPA in the central nervous system has been gradually revealed, with the positive γ-aminobutyric acid A receptor regulating stress and sex hormones such as pregnenolone and MPA, which can lead to cognitive dysfunction (Bäckström et al., 2023; Roden et al., 2020). The use of MPA, particularly in injectable forms, has been associated with an increased risk of intracranial meningioma (Griffin, 2024; Roland et al., 2024), and this association is enhanced with a longer duration of use, which may explain the adverse neurological effects of MPA, offering new possibilities for the treatment of some neurological disorders.

In the product monographs of MPA, elevated blood pressure is widely recognized as an adverse effect (Roland et al., 2024). Notably, our study identified a strong signal for decreased blood pressure, which might be caused by multiple factors, such as individual variations, dosage control, drug interactions, or the specific physiological state of the patient. This discrepancy warrants further investigation to elucidate the underlying mechanisms and to better inform clinical decision-making. Additionally, dental caries emerged as a novel adverse effect in our study, warranting attention. Fluctuations in hormone levels may influence the reactivity of the gum tissue (Heasman and Hughes, 2014), potentially leading to the development of dental caries.

Adverse events associated with MPA exhibited an early failure pattern, with the majority (60.37%) of women experiencing AEs within the first year of contraceptive use. Thereafter, the incidence of AEs decreased over time. The pharmacokinetic properties of this medication may have contributed to the observed variability in the onset of AEs. The robustness of our study is underscored by the comprehensive analysis of a vast dataset encompassing over 21 million AE reports from the FAERS database. Our meticulous methodology, which included the application of multiple statistical tools, such as ROR, PRR, and EBGM, ensures a thorough and reliable assessment of the safety profile of MPA. Furthermore, the time-to-onset analysis provided critical insights into the temporal relationship between MPA use and the manifestation of AEs, reinforcing the validity of our findings. The scale and rigor of our pharmacovigilance analysis offer a solid foundation for clinical decision-making and future research directions in the field of contraceptive safety.

Although informative, this study has some limitations. First, pharmacovigilance analysis utilizing the FAERS database establishes associations between specific drugs and AEs but does not establish causal relationships. Second, The FAERS database is a spontaneous reporting database; as such, there may be underreporting, which could lead to bias caused by reporting. At the same time, uneven quality of reporting personnel, ambiguous and non-standard data may also cause analysis bias. Third, the FAERS database primarily contains reports from the United States, which may limit the generalizability of our findings to other regions, such as China, owing to variations in demographic and regional factors.

## 5 Conclusion

This retrospective study leveraged real-world FAERS data to uncover potential MPA AEs not included in product labeling. This approach helps to address the limitations of pre-market clinical trials and provides a reference for clinical safety in medication use. In the future, larger scale clinical observations and real-world studies are needed to confirm these results and increase the safety of MPA medication.

## Data Availability

The datasets presented in this study can be found in online repositories. The names of the repository/repositories and accession number(s) can be found in the article/Supplementary Material.

## Abbreviations

AEs, Adverse Effects; ADRS, Adverse Drug Reactions; EBGM, Empirical Bayesian geometric mean; FAERS, FDA Adverse Event Reporting System; MPA, Medroxyprogesterone acetate; PRR, Proportional reporting ratio; PT, Preferred term; PS, primary suspect; ROR, Reporting odds ratio; SOC, System Organ Class; TTO, time-to-onset.

## References

[B1] Aderoba A. K. Steyn P. S. Kiarie J. N. (2023). Implementation strategies to scale up self-administered depot medroxyprogesterone acetate subcutaneous injectable contraception: a scoping review. Syst. Rev. 12 (1), 114. 10.1186/s13643-023-02216-2 37403147 PMC10318699

[B2] Armas L. A. G. Recker R. R. (2012). Pathophysiology of osteoporosis: new mechanistic insights. Endocrinol. Metabolism Clin. N. Am. 41 (3), 475–486. 10.1016/j.ecl.2012.04.006 22877425

[B3] Bäckström T. Turkmen S. Das R. Doverskog M. Blackburn T. P. (2023). The GABA system, a new target for medications against cognitive iMPAirment—associated with neuroactive steroids. J. Intern. Med. 294 (3), 281–294. 10.1111/joim.13705 37518841

[B4] Bender R. A. (2022). Medroxyprogesterone acetate for abnormal uterine bleeding due to ovulatory dysfunction: the effect of 2 different-duration regimens. Med. Sci. Monit. 28, e936727. 10.12659/msm.936727 35746846 PMC9248354

[B5] Chauvet-Gelinier J.-C. Bonin B. (2017). Stress, anxiety and depression in heart disease patients: a major challenge for cardiac rehabilitation. Ann. Phys. Rehabilitation Med. 60 (1), 6–12. 10.1016/j.rehab.2016.09.002 27771272

[B6] Chen X. Jiang Y. Zhu H. Tian M. (2024). Mining and evaluation of adverse event signals for capmatinib based on the FAERS database. Front. Pharmacol. 15, 1417661. 10.3389/fphar.2024.1417661 39380910 PMC11458401

[B7] Cundy T. Evans M. Roberts H. Wattie D. Ames R. Reid I. R. (1991). Bone density in women receiving depot medroxyprogesterone acetate for contraception. Bmj 303 (6793), 13–16. 10.1136/bmj.303.6793.13 1830502 PMC1670252

[B8] Dabee S. Balle C. Onono M. Innes S. Nair G. Palanee-Phillips T. (2023). Update on the impact of depot medroxyprogesterone acetate on vaginal mucosal endpoints and relevance to sexually transmitted infections. Curr. HIV/AIDS Rep. 20 (4), 251–260. 10.1007/s11904-023-00662-0 37341916 PMC10403392

[B9] Griffin R. L. (2024). The association between medroxyprogesterone acetate exposure and meningioma. Cancers 16 (19), 3362. 10.3390/cancers16193362 39409982 PMC11482550

[B10] Heasman P. A. Hughes F. J. (2014). Drugs, medications and periodontal disease. Br. Dent. J. 217 (8), 411–419. 10.1038/sj.bdj.2014.905 25342347

[B11] Irigoyen M.-C. De Angelis K. dos Santos F. Dartora D. R. Rodrigues B. Consolim-Colombo F. M. (2016). Hypertension, blood pressure variability, and target organ lesion. Curr. Hypertens. Rep. 18 (4), 31. 10.1007/s11906-016-0642-9 27002717

[B12] Jiang X. Aragaki A. K. Nudy M. Manson J. E. Shadyab A. H. Wild R. A. (2023). The association of hormone therapy with blood pressure control in postmenopausal women with hypertension: a secondary analysis of the Women's Health Initiative clinical trials. Menopause (New York, N.Y.) 30 (1), 28–36. 10.1097/GME.0000000000002086 36256926

[B13] Kanis J. A. Cooper C. Rizzoli R. Reginster J. Y. (2018). European guidance for the diagnosis and management of osteoporosis in postmenopausal women. Osteoporos. Int. 30 (1), 3–44. 10.1007/s00198-018-4704-5 30324412 PMC7026233

[B14] Lopez L. M. Grimes D. A. Schulz K. F. (2019). Steroidal contraceptives: effect on carbohydrate metabolism in women without diabetes mellitus. Cochrane database Syst. Rev., 2019, CD006133(11), 10.1002/14651858.CD006133.pub5 31711271

[B15] Matovu F. K. Nabwana M. Kiwanuka N. Scholes D. Isingel E. Nolan M. L. (2020). Bone mineral density in antiretroviral therapy‐naïve HIV‐1–Infected young adult ‐women using depot medroxyprogesterone acetate or nonhormonal contraceptives in Uganda. JBMR Plus 5 (2), e10446. 10.1002/jbm4.10446 33615111 PMC7872338

[B16] Roden R. C. Noritz G. McKnight E. R. Bonny A. E. (2020). An exploratory study of depot-medroxyprogesterone acetate and bone mineral density in adolescent and young adult womenwith cerebral palsy. Contraception 101 (4), 273–275. 10.1016/j.contraception.2019.12.009 31935387

[B17] Roland N. Neumann A. Hoisnard L. Duranteau L. Froelich S. Zureik M. (2024). Use of progestogens and the risk of intracranial meningioma: national case-control study. Bmj 384, e078078. 10.1136/bmj-2023-078078 38537944 PMC10966896

[B18] Rosenthal M. A. McQuillan S. K. (2021). Adolescent contraception. Can. Med. Assoc. J. 193 (31), E1218. 10.1503/cmaj.202413 34373270 PMC8367427

[B19] Santoro N. Roeca C. Peters B. A. Neal-Perry G. (2021). The menopause transition: signs, symptoms, and management options. J. Clin. Endocrinol. Metabolism 106 (1), 1–15. 10.1210/clinem/dgaa764 33095879

[B20] Shiferaw M. Kassahun W. Zawdie B. (2021). Anthropometric indices, blood pressure, and lipid profile status among women using progestin-only contraceptives: comparative cross-sectional study. BMC Women’s Health 21 (1), 34. 10.1186/s12905-021-01178-8 33485353 PMC7824919

[B21] Shu Y. Ding Y. Dai B. Zhang Q. (2021). A real-world pharmacovigilance study of axitinib: data mining of the public version of FDA adverse event reporting system. Expert Opin. Drug Saf. 21 (4), 563–572. 10.1080/14740338.2022.2016696 34918584

[B22] Sims J. Lutz E. Wallace K. Kassahun-Yimer W. Ngwudike C. Shwayder J. (2020). Depo-medroxyprogesterone acetate, weight gain and amenorrhea among obese adolescent and adult women. Eur. J. Contracept. Reproductive Health Care 25 (1), 54–59. 10.1080/13625187.2019.1709963 PMC8569696 31928370

[B23] Tamauchi S. Nakagawa A. Yoshida K. Yoshihara M. Yokoi A. Yoshikawa N. (2024). Update on the oncologic and obstetric outcomes of medroxyprogesterone acetate treatment for atypical endometrial hyperplasia and endometrial cancer. J. Obstetrics Gynaecol. Res. 50, 1614–1621. 10.1111/jog.16038 39092804

[B24] Teal S. Edelman A. (2021). Contraception selection, effectiveness, and adverse effects: a review. Jama 326 (24), 2507–2518. 10.1001/jama.2021.21392 34962522

[B25] Upadhyay U. D. Zlidar V. M. Foster D. G. (2016). Interest in self-administration of subcutaneous depot medroxyprogesterone acetate in the United States. Contraception 94 (4), 303–313. 10.1016/j.contraception.2016.06.006 27326938

[B26] van Weelden W. J. Birkendahl P. B. Lalisang R. I. IntHout J. Kruitwagen R. F. P. M. Romano A. (2022). The effect of progestin therapy in advanced and recurrent endometrial cancer: a systematic review and meta‐analysis. BJOG Int. J. Obstetrics and Gynaecol. 130 (2), 143–152. 10.1111/1471-0528.17331 PMC10100186 36264251

[B27] Wang L. T. Chen L. R. Chen K. H. (2023). Hormone-related and drug-induced osteoporosis: a cellular and molecular overview. Int. J. Mol. Sci. 24 (6), 5814. 10.3390/ijms24065814 36982891 PMC10054048

[B28] Watts N. B. Binkley N. Owens C. D. Al-Hendy A. Puscheck E. E. Shebley M. (2021). Bone mineral density changes associated with pregnancy, lactation, and medical treatments in premenopausal women and effects later in life. J. Women’s Health 30 (10), 1416–1430. 10.1089/jwh.2020.8989 PMC13174973 34435897

[B29] Xuan G. Zhang Y. Cui J. Zhou J. Sui C. (2023). Propofol-associated serious adverse events: an analysis of the FAERS database. Biotechnol. Genet. Eng. Rev. 40, 2874–2887. 10.1080/02648725.2023.2202541 37066882

[B30] Yu J. H. Moon M. K. Ahn H. C. Yang Y.-M. (2024). Assessing medication use patterns among patients with polycystic ovary syndrome at a tertiary care teaching hospital in South Korea:A retrospective study. Medicine 103 (32), e39055. 10.1097/md.0000000000039055 39121320 PMC11315483

[B31] Yu R. J. Krantz M. S. Phillips E. J. Stone C. A. (2021). Emerging causes of drug-induced anaphylaxis: a review of anaphylaxis-associated reports in the FDA adverse event reporting system (FAERS). J. Allergy Clin. Immunol. Pract. 9 (2), 819–829.e2. 10.1016/j.jaip.2020.09.021 32992044 PMC7870524

[B32] Zhang Y. Zhou C. Liu Y. Hao Y. Wang J. Song B. (2024). Adverse event signal mining and severe adverse event influencing factor analysis of Lumateperone based on FAERS database. Front. Pharmacol. 15, 1472648. 10.3389/fphar.2024.1472648 39376606 PMC11456470

