# Supplementary material for: Safety evaluation of medroxyprogesterone acetate: a pharmacovigilance analysis using FDA adverse event reporting system data
Source: Front Pharmacol. 2024 Dec 11;15:1491032. doi: 10.3389/fphar.2024.1491032 (PMC11701781; doi:10.3389/fphar.2024.1491032)
Supplement: Supplementary file 1 [file Table1.DOCX]

**Supplementary Table 1.** Three major algorithms used for signal detection.

| Algorithms | Equation | Criteria |
| --- | --- | --- |
| Reporting Odds Ratio  (ROR) | ROR=ad/b/c | lower limit of 95% CI>1, N≥3 |
|  | 95%CI=e^ln(ROR)±1.96(1/a+1/b+1/c+1/d)^0.5^ |  |
| Proportional Reporting Ratio (PRR) | PRR=a(c+d)/c/(a+b) | PRR≥2, χ^2^≥4, N≥3 |
|  | χ^2^=[(ad-bc)^2](a+b+c+d)/[(a+b)(c+d)(a+c)(b+d)] |  |
| Multi-item Gamma  Poisson Shrinker  (MGPS) | EBGM=a(a+b+c+d)/(a+c)/(a+b) | EBGM05>2 |
|  | 95%CI=e^ln(EBGM)±1.96(1/a+1/b+1/c+1/d)^0.5^ |  |

Equation: a, number of reports containing both the target drug and target adverse drug reaction; b, number of reports containing other adverse drug reaction of the target drug; c, number of reports containing the target adverse drug reaction of other drugs; d, number of reports containing other drugs and other adverse drug reactions. 95%CI, 95% confidence interval; N, the number of reports; χ^2^, chi-squared; the variance of IC; EBGM, empirical Bayesian geometric mean; EBGM05, the lower limit of 95% CI of EBGM.
